# Design and Implementation of a Lightweight and Energy-Efficient Semantic Segmentation Accelerator for Embedded Platforms

**DOI:** 10.3390/mi16030258

**Published:** 2025-02-25

**Authors:** Hui Li, Jinyi Li, Bowen Li, Zhengqian Miao, Shengli Lu

**Affiliations:** School of Integrated Circuits, Southeast University, Nanjing 211189, China; lihui_666@163.com (H.L.); lijinyi@seu.edu.cn (J.L.); seu17778735086@163.com (B.L.); 220246690@seu.edu.cn (Z.M.)

**Keywords:** lightweight architecture, semantic segmentation, neural network accelerator, FPGA, energy efficient

## Abstract

With the rapid development of lightweight network models and efficient hardware deployment techniques, the demand for real-time semantic segmentation in areas such as autonomous driving and medical image processing has increased significantly. However, realizing efficient semantic segmentation on resource-constrained embedded platforms still faces many challenges. As a classical lightweight semantic segmentation network, ENet has attracted much attention due to its low computational complexity. In this study, we optimize the ENet semantic segmentation network to significantly reduce its computational complexity through structural simplification and 8-bit quantization and improve its hardware compatibility through the optimization of on-chip data storage and data transfer while maintaining 51.18% mIoU. The optimized network is successfully deployed on hardware accelerator and SoC systems based on Xilinx ZYNQ ZCU104 FPGA. In addition, we optimize the computational units of transposed convolution and dilated convolution and improve the on-chip data storage and data transfer design. The optimized system achieves a frame rate of 130.75 FPS, which meets the real-time processing requirements in areas such as autonomous driving and medical imaging. Meanwhile, the power consumption of the accelerator is 3.479 W, the throughput reaches 460.8 GOPS, and the energy efficiency reaches 132.2 GOPS/W. These results fully demonstrate the effectiveness of the optimization and deployment strategies in achieving a balance between computational efficiency and accuracy, which makes the system well suited for resource-constrained embedded platform applications.

## 1. Introduction

Compared to conventional methods, semantic segmentation techniques based on Convolutional Neural Networks (CNNs) have the ability to capture contextual relationships at different scales, enabling a more holistic understanding of semantic information in images [1,2,3]. These methods have emerged as the predominant technique in the field of semantic segmentation and have significant implications in areas such as intelligent security and autonomous driving [4,5]. However, these practical applications require real-time performance and high efficiency in semantic segmentation [6,7]. For example, in video surveillance, semantic segmentation can analyze video streams in real time to detect abnormal behaviors or specific targets, improving monitoring efficiency. Consequently, there has been a surge of research interest in hardware acceleration of CNN-based models [8,9,10,11,12] for semantic segmentation.

Currently, numerous researchers are dedicated to enhancing the performance of semantic segmentation accelerators by exploring parallel convolutional computations. For instance, methodologies cited in references [13,14,15,16,17] have significantly bolstered the computational capabilities of accelerators through the parallelization of convolutional operations. Other literature [18,19,20,21,22] has employed traditional lightweight model designs, quantitative pruning, and other techniques to expedite the model’s inference process. However, the existing literature lacks adequate optimization designs for commonly employed transposed convolutions and dilated convolutions in semantic segmentation networks [23,24,25,26]. For example, Papatheofanous [14] utilized deep learning processing unit (DPU) IP cores, high-level synthesis (HLS) kernels, multithreading, and CPU optimization to accelerate the entire semantic segmentation application [27]. Nevertheless, this approach fails to address the hardware design targeted at the redundant zero computations in transposed convolutions and dilated convolutions [28,29]. The proportion of redundant zero computations exceeds 60% for both types of convolutions, significantly impeding the energy efficiency [30,31]. To tackle these issues comprehensively, we also examine and analyze recent advancements in hardware acceleration for machine learning algorithms. Many surveys of hardware accelerators for deep learning focus on optimizing different parts of the machine learning pipeline, including neural network training, inference, and model deployment [32,33].

In the context of lightweight semantic segmentation, it is crucial to balance computational complexity with hardware constraints, especially when dealing with operations like transposed and dilated convolutions, which have a significant proportion of redundant zero computations. This motivates the need for specialized hardware designs that can efficiently exploit the sparsity in these convolutions and reduce unnecessary computations. Our study focuses on proposing a design methodology that not only optimizes the semantic segmentation model through quantization and network architecture redesign but also thoroughly analyzes the hardware design space, particularly the memory and computation strategies required to optimize the efficiency of transposed and dilated convolutions.

To address the aforementioned issues, a highly energy-efficient accelerator for lightweight semantic segmentation networks is proposed in this paper. The contributions of this work can be summarized as follows:

(1) The semantic segmentation network is quantized to reduce the computational complexity and minimize the loss of recognition accuracy while ensuring compatibility with hardware. The technique facilitates the deployment of the model to hardware accelerators, thereby improving energy efficiency and performance.
(2) A configurable delay-line caching module is proposed to efficiently perform convolution calculations on zero-padded convolution kernels in dilated convolutions. The proposed module enhances the efficiency of dilated convolution computations.
(3) A configurable computation array is devised for efficient computation of transposed convolutions on zero-padded feature maps. The array calculations are reconstructed and distributed to generate multiple accurate and complete outputs of transposed convolution computations.
(4) An optimization design of the input–output buffer module is performed to maximize the acceleration effect of array convolution computations while adapting to different array throughput scenarios for convolution calculations.

The organizational structure of this paper is described as follows: Section 2 systematically analyzes lightweight optimization techniques for semantic segmentation networks. In Section 3, the design methodology of energy-efficient accelerators for lightweight networks is presented, including key hardware modules and optimization strategies. Section 4 reports experimental results, including the performance improvement of the semantic segmentation model based on the proposed methodology and FPGA implementation results. Finally, Section 5 concludes the study and discusses future work.

## 2. Lightweight Semantic Segmentation Network Design

### 2.1. Semantic Segmentation Network Structure Lightweighting

In order to achieve lightweight semantic segmentation, this paper carries out a number of optimization improvements based on the classical ENet (efficient neural network) [8], which significantly reduces the number of parameters and computational complexity while maintaining the accuracy at a level close to that of the original network.

The ENet network adopts a modular design, and the bottleneck module of the encoder consists of downsampling, as well as regular and dilated modules, while the bottleneck module of the decoder consists of upsampling and regular modules. In order to further reduce the weight, the network structure is optimized to explore the possibility of introducing deeply separable convolution. It is observed that the network already contains a dot convolution structure consistent with depth-separable convolution, which provides a basis for introducing depth-separable convolution.

**Figure 1:**
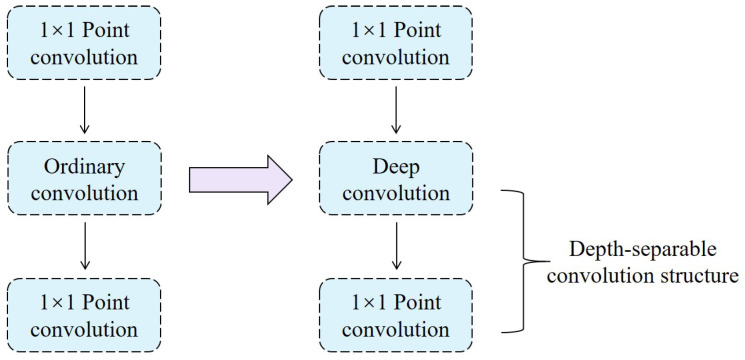
Optimization of network structure.

In order to make the network more lightweight, this paper improves the network structure and explores the possibility of introducing depth-separable convolution. Through analysis, it is found that there is already a point convolution structure in the network that is consistent with depth-separable convolution. Therefore, the applicability of the deep convolution structure is further explored next. Ordinary convolution refers to the standard convolution operation, where each kernel operates across all input channels and generates an output feature map for each channel, while deep convolution processes each input channel independently using separate kernels, significantly reducing computational complexity. The advantage of deep convolution lies in its lower computational cost and reduced number of parameters, as each kernel is smaller and only operates on a single channel. However, this may leads to the problem of underutilization of inter-channel information due to the independent computation of each channel layer. Therefore, deep convolution needs to be followed by concatenated dot convolution to fuse the information between channels. In the bottleneck structure, the 3 × 3 standard convolution of the right branch can be replaced by deep convolution; then, the subsequent point convolution can be connected to form a deep separable convolution, as shown in Figure 1, which reduces the number of network parameters and computational burden.

### 2.2. Quantization of Semantic Segmentation Networks

In terms of quantization, the weights and activation values of the network are 8-bit quantized with reference to the DoReFa-Net algorithm, which circumvents the problem of zero gradient by straight-through estimation (STE) and establishes a functional relationship between the input gradient and the output gradient. Its forward and backward formulas are shown in (1):

(1) $$\left\{\begin{array}{c} Forward:r_{o}=quantize_{k}\left(r_{i}\right)=\frac{1}{2^{k}-1}round\left(\left(2^{k}-1\right)r_{i}\right)\\ Backward:\frac{\partial C}{\partial r_{i}}=\frac{\partial C}{\partial r_{o}} \end{array}\right.$$

where the *k* parameter represents the bit width used for quantization.

Quantization of the network weights is conducted by converting floating-point numbers to *k* bits of fixed-point numbers using the method shown in Formula (2). This process is accomplished by distributing the weight values uniformly within the [−1, 1] range to optimize storage efficiency.

(2) $$\left\{\begin{array}{c} Forward:r_{o}=2quantize_{k}\left(\frac{\tanh\left(r_{i}\right)}{2\max\left(\left|\tanh\left(r_{i}\right)\right|\right)}+\frac{1}{2}\right)-1\\ Backward:\frac{\partial C}{\partial r_{i}}=\frac{\partial C}{\partial r_{o}}\frac{\partial r_{o}}{\partial r_{i}} \end{array}\right.$$

The $r_{i}$ variable denotes the original floating-point weight value, while $r_{0}$ represents the quantized weight value, which is mapped to the range of [−1, 1]. The ${\mathrm{quantize}}_{k}\left(r_{i}\right)$ function is the quantization function that maps the floating-point number ($r_{i}$) to a k-bit fixed-point number.

To maximize the storage efficiency of the weights, this paper optimizes the weight quantization Formula (2) by mapping the weights to the range of of [−1, 1]. The final weight quantization is shown in Formula (3).

(3) $$r_{o}=clip\left(\frac{1}{2^{k}-1}round\left(2^{k-1}\left[2\left(\frac{\tanh(r_{l})}{2\max(|\tanh\left(r_{l}\right)|)}+\frac{1}{2}\right)-1\right]\right),-\frac{2^{k-1}-1}{2^{k-1}},\frac{2^{k-1}-1}{2^{k-1}}\right)$$

For activation quantization, the constraint function (h(x)) is used as shown in Formula (4). The activation value (x) is mapped to the range of [0, 1]. Three commonly used constraint functions include the clip function, which is used to limit the data range.

(4) $$h\left(x\right)=\left\{\begin{array}{c} \frac{tanh(x)+1}{2}\\ clip(x,0.1)\\ min(1,|x|) \end{array}\right.$$

The clip(x,0.1) function is a clipping operation that constrains the value of *x* within a specified range.

In the comparison between hardware and software implementations, software is generally more advantageous for handling complex operations like tanh(x), as it supports high-precision floating-point arithmetic and is easier to modify and optimize. In contrast, hardware (such as FPGA) typically uses fixed-point representation, which limits precision and introduces quantization errors. Specifically, a floating-point range like [0, 1] is difficult to directly implement in hardware, as fixed-point systems cannot accurately represent continuous values. Therefore, software implementation is better suited for operations requiring high precision and flexibility.

## 3. Accelerator Architecture

### 3.1. Overall Hardware System

This paper presents the design of an FPGA (Field-Programmable Gate Array)-based hardware accelerator using the hardware–software collaborative design method, utilizing the process system (PS) and programmable logic (PL) side of the ZYNQ SoC (System on Chip) platform to work together to realize the system design of the accelerator. While Xilinx provides tool chains (e.g., Vitis AI) for deploying neural networks with Deep Processing Units (DPUs), support for custom operations like dilated and transposed convolutions remains limited. This motivates our hardware–software collaborative design approach to optimize these operations specifically for semantic segmentation tasks.

**Figure 2:**
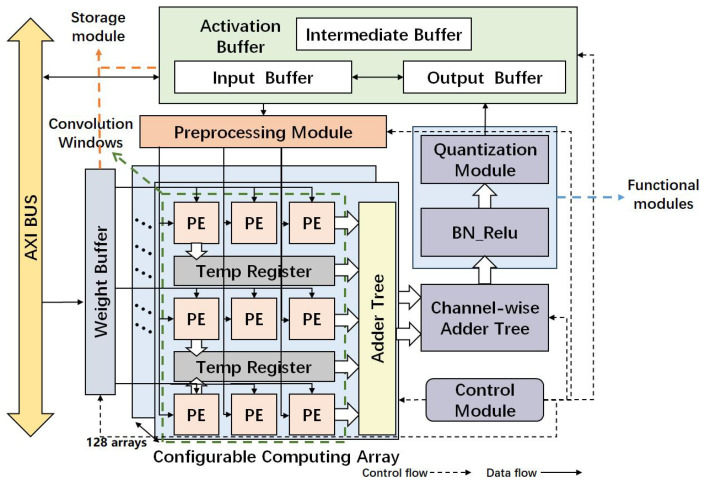
The overall architecture of the proposed accelerator.

Figure 2 illustrates the comprehensive architecture of the accelerator, comprising a control module, pre-processing module, configurable computing array, functional modules for normalized activation, quantization and other functions, storage module consisting of a weight buffer and activation buffer, and AXI interface connected to the PS terminal. The control module is the core unit of the accelerator. It configures various modules of the accelerator based on the type of convolution currently being executed and the size of the input feature maps. It also manages control tasks such as controlling the accelerator to read from the weight buffer and activation buffer, initiating computation and interacting with other modules to switch the accelerator’s operational state. The preprocessing module is primarily used to generate feature-map windows and weight windows for different types of convolutional computations. The configurable computing array consists of 128 sets of 3 × 3 PE arrays. The storage module comprises a weight buffer and activation buffer. The network’s weight and bias parameters are stored in on-chip BRAM during the initialization phase of running the accelerator. Feature maps are also pre-loaded into an on-chip input buffer composed of Ultra-Ram, while intermediate-layer parameters are handled using a PING-PONG operation between two input and output buffers built with Ultra-Ram. Additionally, there is a dedicated intermediate buffer specifically for the processing of residual network structures. Functional modules include computational operations beyond convolutions, such as activation and quantization modules. These functional modules correspond to their respective parts in the semantic segmentation network, ensuring consistency with the implementation of the forward inference process on the software side and enabling the complete deployment of the network.

### 3.2. Accelerator Data Stream

**Figure 3:**
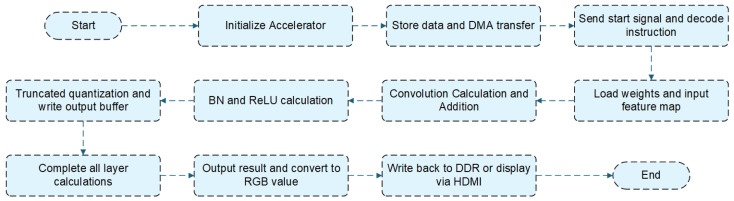
Flowchart of accelerator data stream.

The data stream of the entire accelerator is shown in Figure 3. The overall workflow of the accelerator starts with the initialization of the accelerator from the processor system side (PS side). Subsequently, the accelerator instruction data, weight parameters, BN (Batch Normalization) parameters, and input feature maps corresponding to the running network are stored in specific positions in the DDR buffer on the PS side. DMA (Direct Memory Access) is used to transfer the aforementioned data stored in the DDR buffer on the accelerator to their respective positions. For instance, the weight parameters are transferred to the weight buffer, and the accelerator instructions are stored in the instruction buffer of the control module. Thereafter, a start signal is dispatched to the accelerator, prompting the control module to initiate the forward inference procedure in accordance with the operational paradigm of the network’s inaugural layer. Then, the start signal is sent to the accelerator, initiating the forward inference process controlled by the main controller according to the operational mode of the network’s first layer.

The accelerator control module decodes the instruction data for each layer, obtaining control signals that are sent to various modules for control. The pre-processing module, based on the received control signals, begins by loading the weights and input feature maps. It retrieves the weight data for the corresponding 128 channels from the weight buffer and generates weight values for the respective convolution windows. Additionally, it reads the input feature maps from the input buffer and generates feature-map values for the convolution windows, which are then sent to the PE array. The convolution windows are multiplied, and the results are fed into the addition tree module for inter-array and inter-channel addition. The BN and ReLU modules perform the corresponding calculations based on BN and ReLU operations, respectively. Finally, after truncation quantization, the results are sent back to the output buffer. This sequence of operations is repeated as required. After completing the computation for the current layer, the control module retrieves the instructions for the next layer and starts sending control signals based on the decoded instruction information. This process continues until all inference layers have been computed.

After completion, the final output result of the network is 480 × 360 × 12 × 8 bits, and the prediction conditions for the corresponding pixels are determined by selecting the maximum value. Then, the output is transformed into three-channel RGB values for the respective categories of pixels and either written back to DDR or displayed through the HDMI interface for the visualization of predicted images.

### 3.3. Optimization of Dilated Convolution and Transposed Convolution

#### 3.3.1. Computational Optimization and Row Cache Design for Dilated Convolution

Dilation convolution is commonly used in the encoder part of semantic segmentation networks to capture a wider range of semantic information. It expands the sensory field by inserting voids in the convolutional kernel but, at the same time, introduces a large number of zero-valued redundant computations, leading to computational inefficiency. In order to solve this problem, this paper designs a configurable delayed row-caching module for dilation convolution, which constructs an efficient convolutional sliding window adapted to cavity convolution by effectively handling discontinuities in row and column directions.

**Figure 4:**
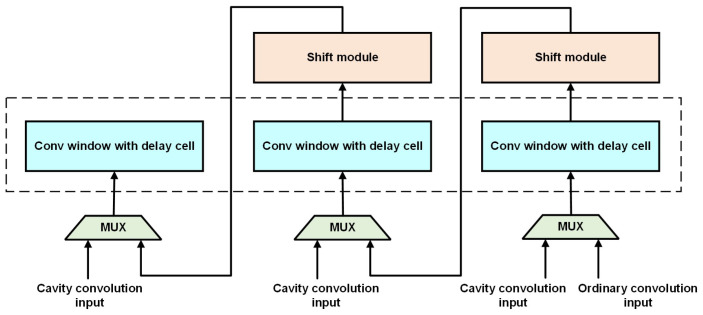
Schematic design for dealing with discontinuities between dilation convolution columns.

To deal with the discontinuities in the row direction, the delayed row-caching module introduces a delay unit in the sliding-window generation process and dynamically configures its depth to match the dilation rate. When the dilation rate is *r*, the depth of the delay unit is set to r−1, which is used to temporarily store unused data and reuse them in the next row of convolutional window generation, avoiding repeated reading of row data, thereby improving data utilization and reducing the pressure on storage bandwidth. As shown in the Figure 4, in response to the discontinuity of data in the column direction, the delayed row-caching module skips the unwanted column data by means of a multiplexer (MUX) and specific logic. For example, when the dilation rate is r=2, only data with even column numbers are selected to participate in the sliding-window operation, which reduces invalid computations and improves the efficiency of column-direction data processing.

**Figure 5:**
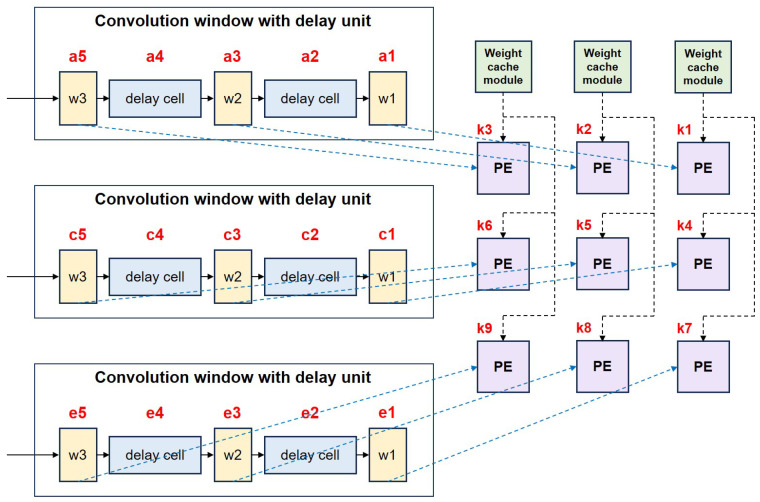
Schematic representation of optimized row-caching convolution sliding window for dilation convolution.

Dilation convolution uses a new design scheme in order to reduce register usage when computing discontinuities between rows. In the process of line caching to the accelerator input feature map cache, a separate cache logic and address generation module (addr_gen) are added. Each address generation module generates a specific fetch address based on the dimensions and dilation rate of the input feature map and feeds the corresponding data into the convolutional window. Specifically, the input to the first layer of the convolution window comes from the start address of the feature map, the input address of the second layer is the start address plus the product of the dilation rate and the width of the feature map, and the input address of the third layer is the start address plus twice the dilation rate multiplied by the width of the feature map. Subsequently, the addresses of each layer are taken one by one in self-incrementing mode to complete the data loading and meet the demand of convolutional computation. This design effectively reduces the use of registers and ensures the smoothness of the convolution process.The final value obtained in the line cache convolution window is shown in Figure 5.

By skipping the computation of the expanded zeros in the convolution kernel, the optimized design significantly reduces the computation. The use of a convolutional kernel with the expansion of the dilation rate increases the amount of computation, but the expansion of the zero-value calculation in the convolutional kernel is actually an invalid calculation. In the optimized design, with a dilation rate of 2, the amount of computation is reduced by 64%, while with a dilation rate of 4, the amount of computation is reduced by about 88%. With an increased dilation rate, skipping of the zero-value calculation increases, an the amount of computation is reduced by a greater magnitude.

#### 3.3.2. Configurable Computational Array Design for Transposed Convolution

Transposed convolution is commonly used in the decoder part of semantic segmentation networks to achieve upsampling of the feature maps by interpolating zeros. However, this zero-insertion operation inserts zeros in and around the middle of the input feature map, leading to a large number of invalid computations. To optimize this process, the computational array designed proposed in this paper adopts a dynamic scheduling strategy, which is optimized for the convolution operations of odd and even rows, respectively, by controlling the input data stream and the computational path of the array so that each convolutional sliding window only computes the valid part corresponding to the non-zero values. Meanwhile, in the hardware implementation, a staging module is added to store some intermediate computation results and combine them with the subsequent computation results, thereby reconfiguring the multiple-transposition convolution operation into a single computation array task, which significantly improves the effective utilization of the array. A 3 × 3 PE array is used in the design, which can simultaneously output the calculation results of multiple convolutional sliding windows in one cycle under the condition of ensuring the full-load operation of the array, which significantly reduces the calculation time and power consumption of the transposed convolution. In addition, with the data storage module, multiple Bank RAMs and optimized data storage methods are used to ensure efficient data inflow and output of results from the computational array, further enhancing the overall performance of the system.

### 3.4. Design of the Pre-Processing Module

The pre-processing module consists of a feature map read-state machine, a weight read-state machine, a line buffer module, and a weight window generation module.

The line buffer module primarily functions to fetch data from the input buffer and generate the corresponding convolution window required for convolutional calculations.

**Figure 6:**
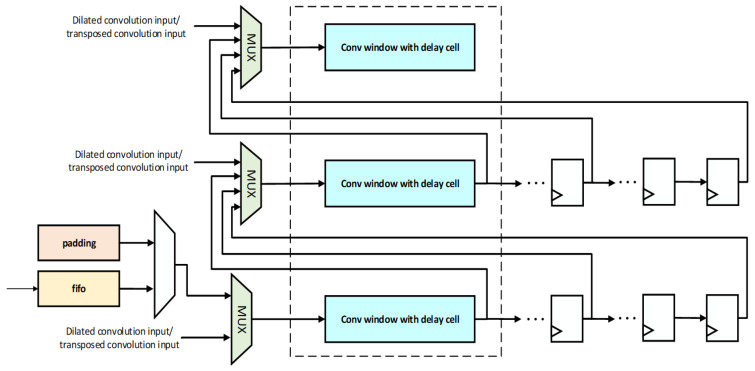
Overview of line buffer module.

The generated window is then fed into the PE array for convolutional computations. It has 64 line buffers and supports configurable convolution window generation for 3 × 3, 2 × 2, and point convolutions. Its structure is illustrated in Figure 6. The padding module pads the input feature map with zeros, corresponding to the positions around the feature map when performing convolution operations with padding. Additionally, with a focus on high energy efficiency, this paper designs a convolutional window with delay units specifically tailored for dilated and transposed convolutions. A multiplexer (MUX) is used to choose between conventional convolution, dilated convolution, and transposed convolution data. Since the input image dimensions may vary, the MUX selects different values from shift registers to be inputted to the convolution window in the next row. Currently, the line buffer configuration can be adjusted according to the image size.

**Figure 7:**
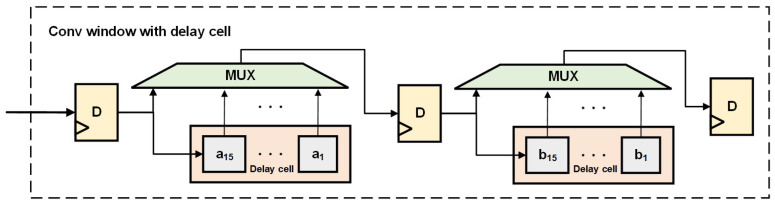
Overview of convolution window with delay cell.

The specific design of the convolution window with delay units is shown in Figure 7. As the maximum dilation factor of dilated convolution is usually 16, 15 delay registers are set in the delay units. Depending on whether it is dilated convolution and the specific value of the dilation factor, the corresponding data are selected using an MUX.

**Figure 8:**
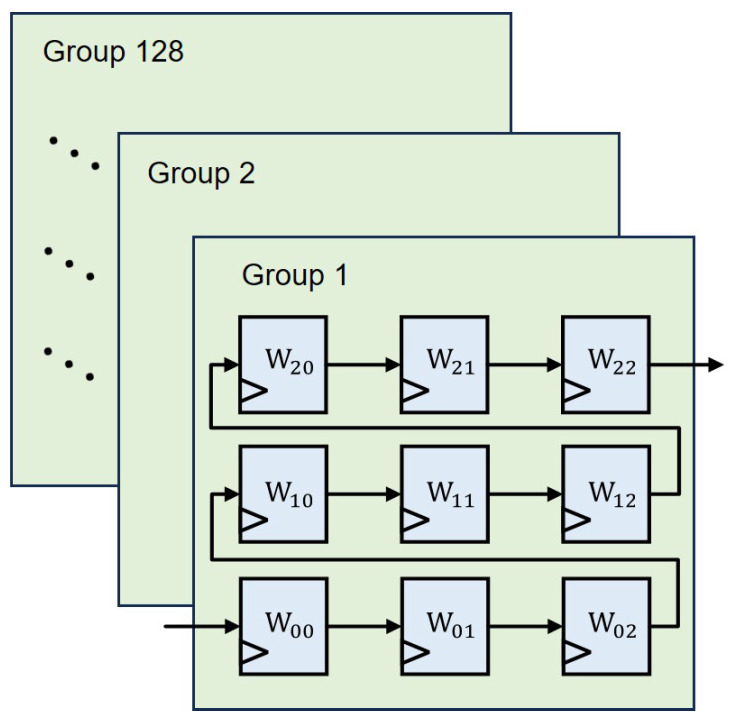
Overview of weight window generation module.

The weight window generation module, as depicted in Figure 8, comprises 128 sets of 9 shift register groups. Upon the input of weight data, the generation of a 3 × 3 convolutional sliding window for weight data is completed after nine cycles, once the initial input array reaches position W_22_. The control of weight data loading for all convolutional scenarios is governed by the weight read-state machine.

The main function of the weight loading-state machine is to control the data transfer from the weight data buffer module to the weight window generation module. Upon receiving the instruction to reload the weights, it begins the weight read process for the corresponding weight buffer address. Upon completing the reading of 9 sets of 128 channels of data (each of the 128 channels comprising 1024 bits), the weight buffer is loaded.

**Figure 9:**
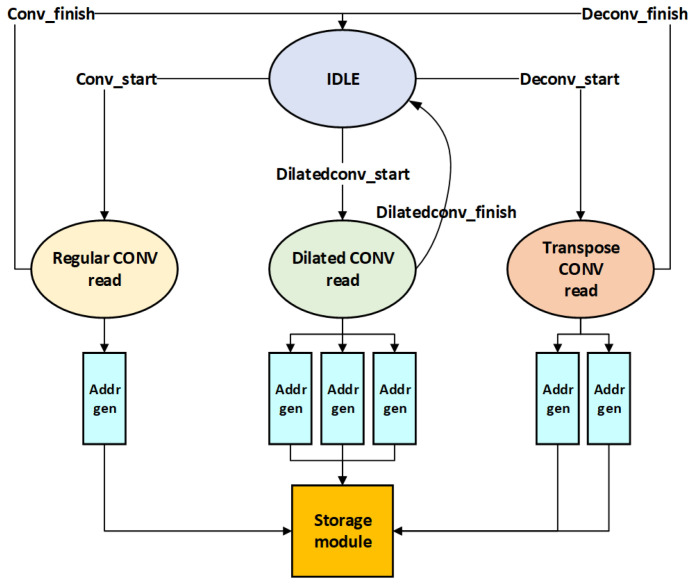
Overview of feature map read-state machine.

The main function of the feature map read-state machine is to control the data transfer from the input data buffer module to the line buffer. The design is relatively complex, as it involves different scenarios, such as regular convolution, depthwise convolution, point convolution, and single-address generation for value retrieval, as illustrated in Figure 9. For dilated convolutions, three address generation (addr_gen) modules are required, while two address generation modules (addr_gen) are needed for transposed convolutions. To minimize hardware overhead, two of the address generation modules used for dilated convolutions are reused. When the state machine receives the start signal, the data retrieval type is determined based on the convolution type.

Upon receipt of the initiation signal, the state machine subsequently discerns the type of data to be read based on the convolution type. If the convolution type is classified as standard convolution, depthwise convolution, or pointwise convolution, the state machine initiates the standard convolution type to perform data reading at a single address. In the case of dilated convolution, the state machine activates the data reading method specific to dilated convolution. Three address generation modules (addr_gen) based on the current dilation coefficient and the dimensions of the feature map generate indices corresponding to the three respective positions of the feature map for dilated convolution. Specifically, the first address index corresponds to the starting address of the feature map, the second address index is the starting address of the feature map plus the dilation coefficient multiplied by the width of the feature map, and the third address index is the starting address of the feature map plus twice the dilation coefficient multiplied by the width of the feature map. For transposed convolution, two address generation modules based on the current dimensions of the feature map generate two indices corresponding to the two respective positions of the feature map for transposed convolution. The first address index corresponds to the starting address of the feature map, and the second address index is the starting address of the feature map plus the width of the feature map.

### 3.5. Design of the Computing Array Module

**Figure 10:**
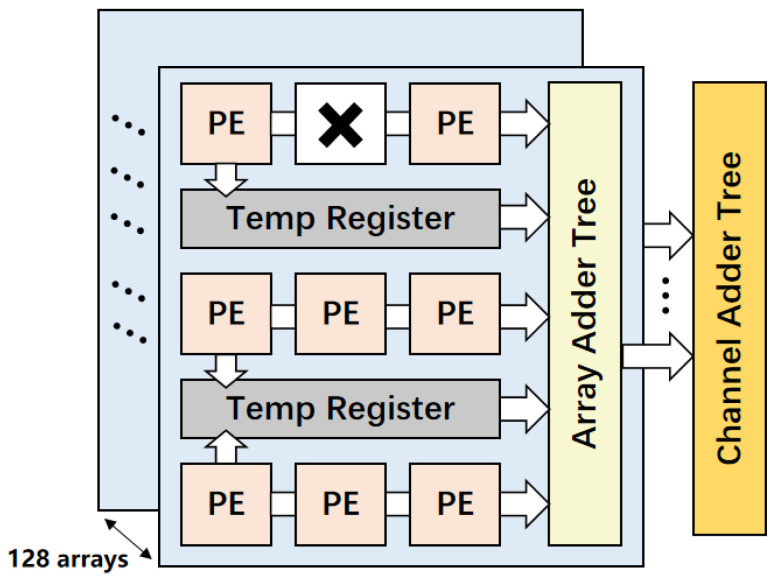
Overview of configurable computing array.

The computation array utilized in this design is illustrated in Figure 10. Each layer of the PE array consists of nine multiplication units corresponding to a conventional convolution window size of 3 × 3. There are a total of 128 layers in the PE array, with each PE housing a single multiplication unit connected to an adder tree module. Temp registers are present between each row of PEs to temporarily store the computed results from the PEs, specifically for the transposed convolution computation mode.

**Figure 11:**
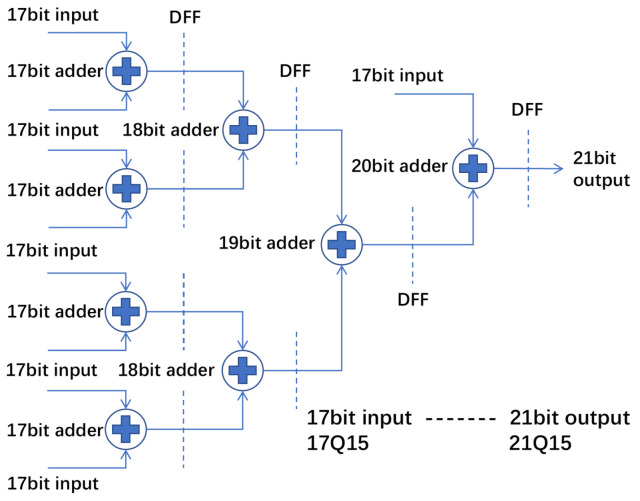
Overview of the array adder tree.

The design of the adder tree module is divided into an array adder tree and channel adder tree. The structure of the array adder tree is shown in Figure 11. The bit-width design of the addition tree is based on the comprehensive consideration of a quantization strategy and dynamic range protection. Since the input feature maps and weights are quantized using 8-bit quantization, the bit width of the multiplication result is 16 bits.To avoid the risk of overflow during the accumulation process, the multiplication result is extended to 17 bits and further accumulated in the addition tree. For the 9-multiplication accumulation of the 3 × 3 convolution kernel, the output bit width of the addition tree is extended to 21 bits to ensure that the maximum possible accumulation values are covered. This design effectively balances the hardware resource overhead while ensuring computational accuracy. The 17-bit signed multiplication result is output from the PE and sent into the array adder tree for addition operations. The 17-bit data are in fixed-point form, with the lowest 15 bits actually representing the fractional part. The dashed lines in the figure represent the registers at each level. After the final 17-bit input, a 21-bit output result is produced. This array adder tree is only suitable for standard convolution, deep convolution, and dilated convolution. Dilated convolution does not require an array adder tree and can be directly sent to the channel adder tree. The channel adder tree is similar to the adder tree. The channel adder tree outputs at different positions of its structure based on the configured number of input channels, with a maximum support for 128 channels to be added together. It supports internal data pipelining.

**Figure 12:**
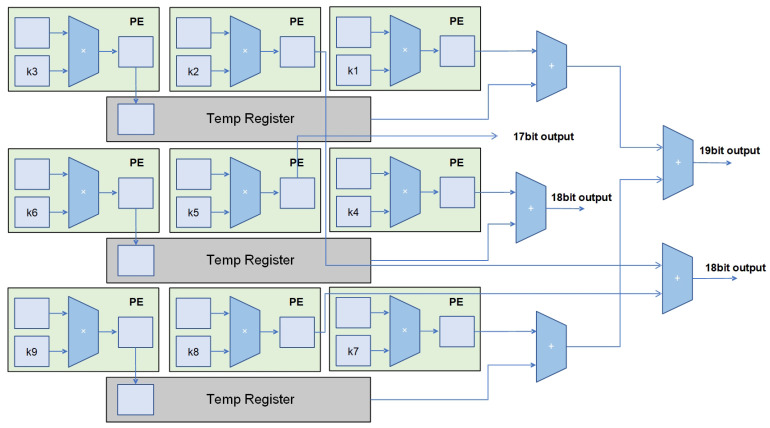
The input–output situation of the array addition tree when running transposed convolution.

For transposed convolution, an additional array adder tree is required, as depicted in Figure 12. Following a design approach optimized for transposed convolution calculations, several extra adders are incorporated to sum the computation results from the array with the values stored in the temp registers. This yields the convolution computation results within the array. In one cycle, a layer of the PE array can output up to four transposed convolution computation results, which are then fed into the inter-channel adder tree for addition calculations.

**Figure 13:**
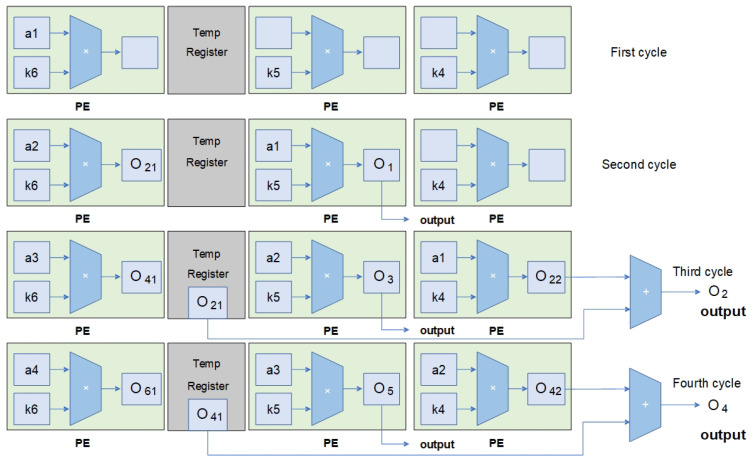
The input–output situation of the second row of PE arrays during transposed convolution.

During transposed convolution operations, the second row of the PE array operates as demonstrated in Figure 13 to illustrate the operation of a single row of PEs. Input feature map data a1∼a4 are sequentially inputted into the PE array. In the second cycle, the computed $O_{1}$ is the output result for the first feature map, and $O_{21}$ is a partial result of the second feature map that is temporarily stored in the temp register. In the third cycle, another partial result ($O_{22}$) of the second feature map is computed and added to the partial sum in the temp register, resulting in the complete output ($O_{2}$) for the second feature map. At this point, outputs $O_{3}$ and $O_{41}$ for the third and fourth feature maps, respectively, are also obtained. This process continues, and each subsequent cycle can output two complete feature map results.

**Figure 14:**
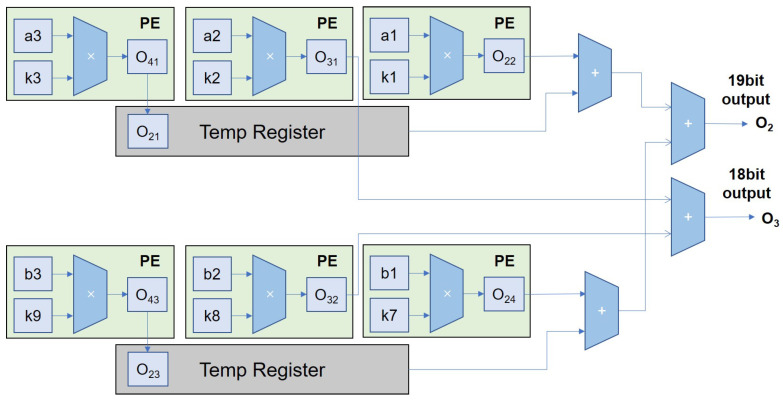
The input–output situation of the first and third rows of PE arrays during transposed convolution.

The corresponding operation of the first and third rows of the PE array is shown in Figure 14. In the third cycle, the complete result for the second output feature map ($O_{2}$) is obtained by summing $O_{21},O_{22},O_{23}$, and $O_{24}$. The third output feature map ($O_{3}$) is obtained by adding $O_{31}$ and $O_{32}$. In each subsequent cycle, two output feature maps can be obtained. Therefore, in a 3 × 3 PE array, four output feature maps can be obtained per cycle, resulting in an efficiency close to four times the original.

### 3.6. Storage Module

Through the evaluation of the overall architecture of the accelerator using the roofline model, the bandwidth requirements for the storage module are determined in order to better leverage the computational power of the computing array. To minimize the number of accesses between the off-chip DDR modules, a significant number of buffer units were designed on the chip, making full use of the storage resources available on the FPGA board. The main buffer modules consist of the network parameter buffer, input/output feature map buffer, and intermediate buffer.

The network parameter buffer consists of the weight buffer and BN-layer parameter buffer. The weight data are entirely stored and processed on the chip, with a width of 1024 bits. A width of 1024 bits allows for the reading of 8-bit weight values for 128 channels in a single operation, which are then input to the weight window generation module. When the number of channels is less than 128, zero padding is applied to the weight parameters before storing them, expanding their width to 1024 bits. The parameter buffer width for the BN layer is 2048 bits, enabling the simultaneous retrieval of 16-bit gamma and 16-bit beta parameters for 64 channels, which are then sent to the BN_Relu module for computation.

**Figure 15:**
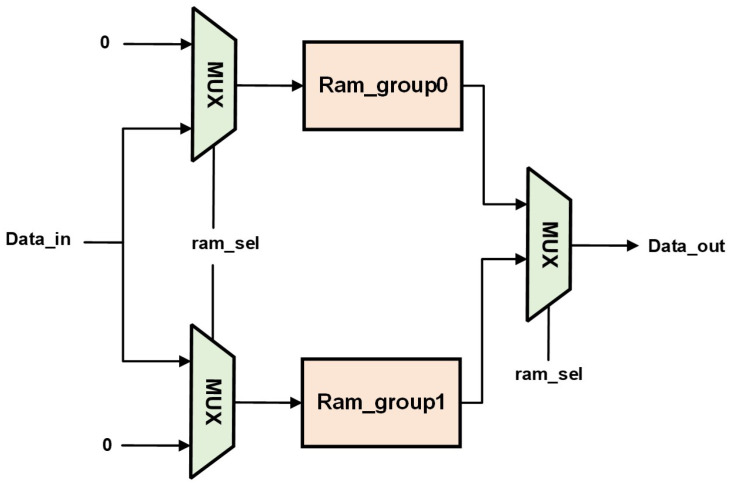
Switching of input and output buffers.

The design of the feature map buffer area primarily consists of three identical storage modules: an input buffer, an output buffer, and an intermediate buffer. After each convolutional layer computation is completed, there is a switch between the input buffer and the output buffer. For example, in Figure 15, if the first layer’s network buffer group 0 (Ram_group0) serves as the input buffer and buffer group 1 (Ram_group1) serves as the output buffer, then in the second layer, Ram_group1 becomes the input buffer, and Ram_group0 becomes the output buffer, alternating the buffering process. The intermediate buffer is utilized to store the branch data of the residual network. In the final convolutional result of the main branch after multiple convolution operations, the output data are added to the data stored in the intermediate buffer to obtain the final result, which is then written into the last buffer group.

**Figure 16:**
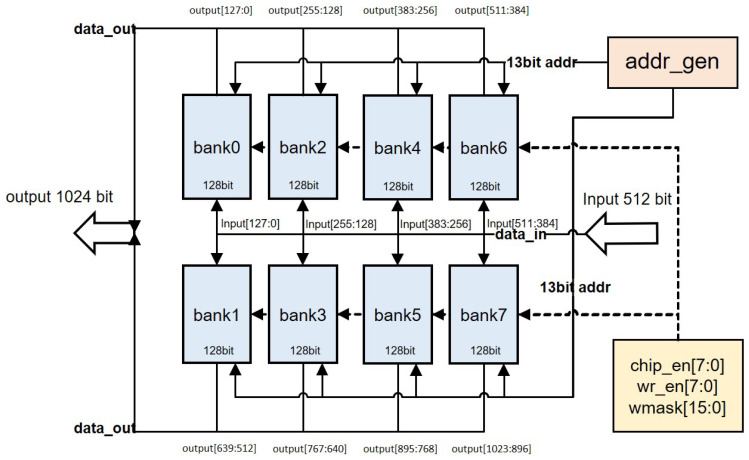
Internal structure diagram of the buffer group.

Each buffer group has an input width of 512 bits and an output width of 1024 bits, as illustrated in Figure 16. Internally, it consists of eight banks, with each bank acting as a pseudo-dual-port RAM. The input and output widths are 128 bits, and the depth is 5400, indicating that each bank can process input and output data for 16 channels of 8-bit data in a single operation. The write and read addresses are controlled by the address generation module (addr_gen).

## 4. Experimental Results and Analysis

In terms of semantic segmentation algorithms, this paper proposes a lightweight semantic segmentation algorithm that leverages a lightweight structure and model quantization methods. By sacrificing a small amount of mIoU, the algorithm significantly reduces the number of model parameters, computational complexity, and data access requirements, making it highly suitable for deployment on FPGA platforms. CamVid is a widely used semantic segmentation dataset consisting of high-resolution images captured in real-world driving scenarios. The dataset provides pixel-wise annotations for various semantic categories, making it an excellent benchmark for evaluating the performance of semantic segmentation algorithms, particularly in real-time applications. We conducted tests on the CamVid dataset, and the performance is summarized in Table 1.

**Figure 17:**
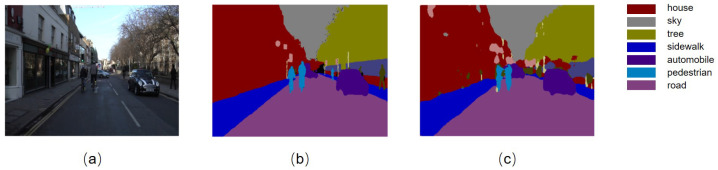
Lightweight semantic segmentation model test image. (**a**) Original image; (**b**) labeled image; (**c**) 8-bit quantized lightweight network recognition result.

Additionally, an example Figure 17 is included to demonstrate the segmentation results of the proposed method on the CamVid dataset, further validating its practical value and efficiency.

The proposed accelerator design is implemented on a ZCU104, a high-performance FPGA development board based on Xilinx’s Zynq UltraScale+ MPSoC chip. The FPGA chip used in this design is the ZYNQ XCZU7EV-2FFVC1156, which features 230 K lookup tables, 11 Mb of BRAM with 27 Mb of Ultra-RAM, 1728 DSP slices, and 461 K flip-flops. The neural network model used in the experiment is ENet.

The proposed accelerator is implemented using a combination of RTL and HLS (High-Level Synthesis) designs. Key modules, such as the configurable computing array and delay line buffer, are developed using Verilog for fine-grained control over hardware resources, enabling precise optimization of critical paths and resource utilization. On the other hand, the preprocessing and control modules are synthesized using Xilinx Vitis HLS, which allows for rapid prototyping and efficient handling of high-level algorithmic transformations. This hybrid approach leverages the strengths of both methodologies: The RTL design provides the flexibility to optimize complex operations like transposed and dilated convolutions, while HLS accelerates the development of control logic and data flow management. The choice of Verilog for the computing array and delay-line buffer is driven by the need to minimize latency and maximize parallelism in these computationally intensive modules. For example, the configurable computing array supports dynamic scheduling of transposed convolution operations, which is not natively supported by off-the-shelf tools.

**Figure 18:**
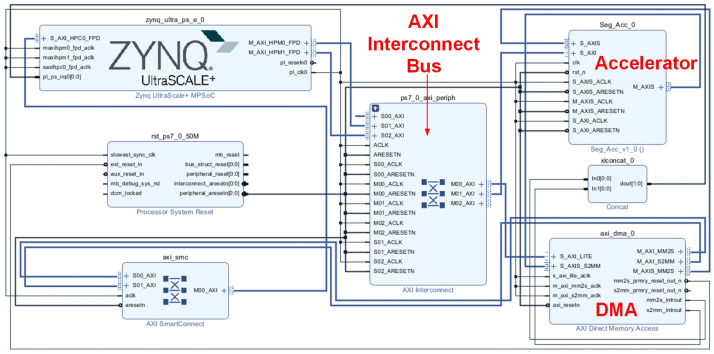
System block design diagram.

The block design diagram of the system is shown in Figure 18, in which the Seg_Acc module is the designed semantic segmentation accelerator module, which is encapsulated into an IP, leaving interfaces with external modules. We can see that there are mainly three interfaces: S_AXIS is the AXI_Stream slave interface, S_AXI is the AXI_Lite slave interface, and M_AXIS is the AXI_Stream master interface. In the upper-left corner is the ZYNQ module, which represents the PS side of the system and is connected to the three corresponding interfaces of the accelerator, and in the lower right corner is the DMA module, which is used for data handling. The PS side uses the AXI_Lite bus to transmit configuration and control information to the accelerator and the AXI_Stream bus to send input feature maps, weights, and batch normalization layer parameters to the accelerator. The accelerator processes the data and transmits the final results back to the PS side via the AXI_Stream bus. The system, as a whole, uses AXI Interconnect for host–slave data interaction.

The overall functionality simulation is carried out by writing a test bench to simulate the entire accelerator. The various modules of the accelerator are integrated into the top-level file according to the designed architecture. Input feature maps, weight parameters, and BN parameters are stored in .txt files. In the test bench, Verilog’s $readmemh function is used to load the input, weight, and BN buffers. Then, the CPU is simulated on the PS side, sending configuration information and start commands to the accelerator. The accelerator begins inference from the first layer of the network and continues layer by layer according to the instruction set until the deployed lightweight semantic segmentation network completes its execution on the accelerator.

**Figure 19:**
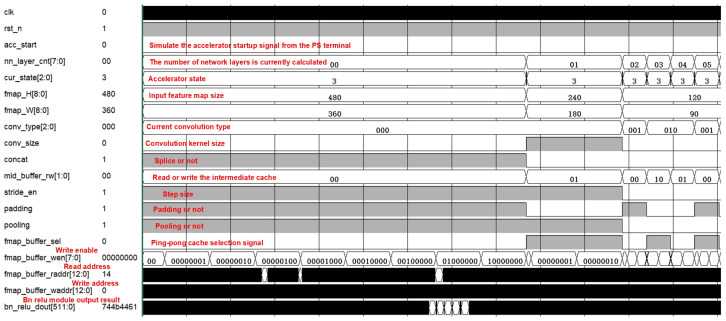
Overall functional simulation diagram.

The overall functional simulation of the accelerator is shown in Figure 19, which illustrates the simulation of the first six layers of the network, and it can be seen that the network size is changed from 360 × 480 to 90 × 120 after two downsampling operations.This is the encoder part of the front section of the semantic segmentation network, and the type of convolution is categorized as standard convolution, point convolution, or deep convolution, as indicated by the signal conv_type. Furthermore, the input cache and output cache are swapped after each layer of computation, using fmap_buffer_sel for selection.

**Figure 20:**
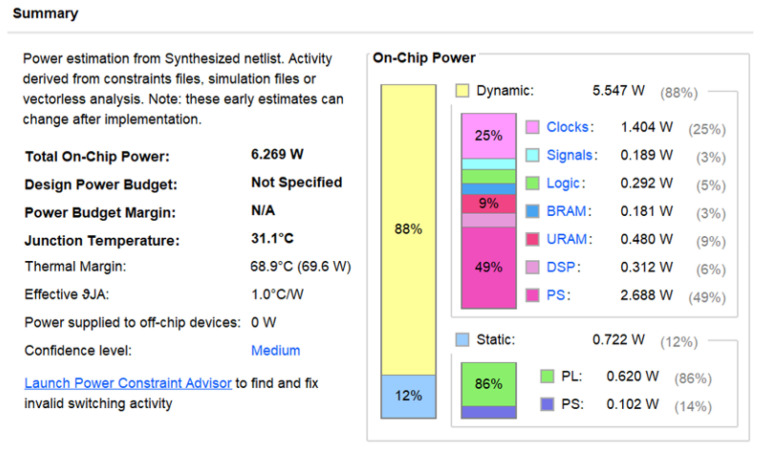
Overall accelerator power consumption.

Table 2 shows the resource utilization of the accelerator system after synthesis. Table 3 presents a comparison of the performance metrics of this accelerator with data from the literature. In terms of power consumption, the accelerator consumes only 3.48 W, while the overall system power is 6.269 W (refer to Figure 20 for specific power consumption data). Based on the peak throughput, the energy efficiency is calculated to be 132.2 GOPS/W, and the average throughput energy efficiency is 100.41 GOPS/W. These values are greater than those reported in the literature, demonstrating the high energy efficiency of this design. In terms of performance, the accelerator achieves a frame rate of 130.75 FPS (Frames Per Second) for forward inference on input images with a size of 360 × 480. This frame rate is higher than results reported in the literature, indicating real-time performance.

## 5. Conclusions

This paper proposes an accelerator for a lightweight semantic segmentation algorithm, efficiently performing standard, pointwise, depthwise, transposed, and dilated convolutions. The design incorporates quantization techniques to reduce memory usage and computational overhead while maintaining segmentation accuracy. The results show that quantization significantly improves performance and energy efficiency compared to traditional methods. A configurable delay line buffer module achieves efficient void convolutional computation, which reduces the computation by 64% to 99.17% with different void coefficients, greatly optimizing the computational efficiency. For transposed convolution, we designed a configurable computational array for convolution computation after zero filling of the feature map. By reconstructing the computation results and distributing them appropriately, multiple correct and complete transposed convolution outputs are obtained, which reduces the amount of redundant computation by 66.67% and improves the computation speed by approximately four times. The input–output buffer module is optimized for varying throughput requirements, maximizing acceleration efficiency. Simulation results show a theoretical average throughput of 348.41 GOPS, with computation efficiencies of 93.11% for dilated convolution and 93.13% for transposed convolution. Overall, the accelerator achieves 75.61% efficiency. At 200 MHz, the accelerator achieves an average throughput rate of 346.81 GOPS, a peak throughput rate of 460.8 GOPS, and a power consumption of 3.48 W, with an energy efficiency of 100.41 GOPS/W. It achieves 130.75 FPS for an input size of 480 × 360, meeting real-time requirements. In summary, by integrating quantization and custom hardware modules, this accelerator balances high performance and low energy consumption, significantly reducing computational redundancy. It outperforms traditional software-based solutions and is suitable for resource-constrained environments.

## Figures and Tables

**Table 1:** Semantic segmentation recognition accuracy comparison.

Reference	[8]	[25]	[34]	Our Work
CNN model	ENet	SegNet-Basic	SegNet	Lightweight ENet
Data width	32bit	8 bit	8 bit	8 bit
Image size	480 × 360	480 × 360	480 × 360	480 × 360
Dataset	CamVid	CamVid	CamVid	CamVid
mIoU	51.3%	46.30%	44.11%	51.18%

**Table 2:** Resource utilization in ZCU104.

Resource	Total	Used	Utilization
BRAM (36 Kb)	11 Mb (312)	89	28.53%
Ultra-RAM (288 Kb)	27 Mb (96)	96	100%
DSP	1728	1262	73.03%
LUT	230 K	178,578	77.51%
Flip Flop	461 K	244,897	53.15%

**Table 3:** Comparison of experimental results.

Reference	[13]	[21]	Our Work
Platform	Xilinx FPGA ZU9	Xilinx FPGA ZU9	Xilinx ZYNQ ZCU104
Frequency	333 MHz	100 MHz	200 MHz
RAM	9.84 Mb	20.21 Mb	BRAM:3.13 Mb URAM:27 Mb
DSPs	-	390	1262
Data width	8 bit	8 bit	8 bit
Performance	1158 GOPS	78 GOPS	460 GOPS
Power consumption	14.5 W	1.2 W	3.48 W
Efficiency	79.9 GOPS/W	65.0 GOPS/W	132.2 GOPS/W
Image input size	480 × 352	300 × 225	480 × 360
Frame rate	128 fps	165.4 fps	130.75 fps

## Data Availability

Data will be made available on request.
